# A study of RNA-editing in *Populus trichocarpa* nuclei revealed acquisition of RNA-editing on the endosymbiont-derived genes, and a preference for intracellular remodeling genes in adaptation to endosymbiosis

**DOI:** 10.48130/FR-2021-0020

**Published:** 2021-11-19

**Authors:** Yiran Wang, Lihu Wang, Su Chen, Song Chen

**Affiliations:** 1 State Key Laboratory of Tree Genetics and Breeding, Northeast Forestry University, Harbin 150040, China; 2 School of Landscape and Ecological Engineering, Hebei University of Engineering, Handan 056000, China

**Keywords:** Nuclear RNA-editing, *Populus trichocarpa*, Endosymbiosis, Endosymbionts, and Organelles

## Abstract

RNA-editing is a post-transcriptional modification that can diversify genome-encoded information by modifying individual RNA bases. In contrast to the well-studied RNA-editing in organelles, little is known about nuclear RNA-editing in higher plants. We performed a genome-wide study of RNA-editing in *Populus trichocarpa* nuclei using the RNA-seq data generated from the sequenced poplar genotype, 'Nisqually-1'. A total of 24,653 nuclear RNA-editing sites present in 8,603 transcripts were identified. Notably, RNA-editing in *P. trichocarpa* nuclei tended to occur on endosymbiont-derived genes. We then scrutinized RNA-editing in a cyanobacterial strain closely related to chloroplast. No RNA-editing sites were identified therein, implying that RNA-editing of these endosymbiont-derived genes was acquired after endosymbiosis. Gene ontology enrichment analysis of all the edited genes in *P. trichocarpa* nuclei demonstrated that nuclear RNA-editing was primarily focused on genes involved in intracellular remodeling processes, which suggests that RNA-editing plays contributing roles in organellar establishment during endosymbiosis. We built a coexpression network using all C-to-U edited genes and then decomposed it to obtain 18 clusters, six of which contained a conserved core motif, A/G-C-A/G. Such a short core motif not only attracted the RNA-editing machinery but also enabled large numbers of sites to be targeted though further study is necessary to verify this finding.

## INTRODUCTION

RNA-editing is a post-transcriptional modification of RNA molecules transcribed from organellar or nuclear genome sequences^[1]^. Such an event may generate proteins that are different from the proteins encoded by the genomic DNA. In humans, thousands of RNA-editing sites have recently been identified in the nuclear genome and the most prevalent editing type is adenosine-to-inosine, A-to-I (G) editing^[2]^. In plants, the research of RNA-editing has been primarily focused on the genomes of mitochondria and chloroplasts^[3,4]^, where RNA-editing preferentially targets the first and second position of codons, which often change the identities of the encoded amino acid^[4]^. As reported, organellar RNA-editing tends to restore the amino acids that are phylogenetically conserved^[4]^. However, there are some exceptions where RNA-editing creates new single amino-acid polymorphisms rather than converting the targeted nucleotides back to the phylogenetically conserved ones^[5]^. The change of an individual amino acid in a protein through RNA-editing can alter a proteins three-dimensional structure and leads to variant functions^[6−8]^. In some rare cases, RNA editing can also generate start or stop codons, resulting in proteins with different or no functions^[5]^. In many other cases, RNA-editing produces synonymous modifications that do not alter protein sequences.

Present studies have revealed that RNA-editing is present in the plant genome, and the nuclear RNA-editing genes are mostly associated with chloroplast and mitochondrion-related functions^[9,10]^. Identification of RNA-editing in nuclei is much more challenging than organelles by virtue of the complexity arising from more RNA species and much larger and a more complicated genome to align to identify the edited sites. Nevertheless, the advent of next-generation sequencing and alignment tools have made it possible to carry out large-scale alignment of terabyte reads, enabling identifying RNA-editing sites on a genome-wide scale.

Plant organelles, as represented by mitochondria and chloroplasts, were evolved from engulfed prokaryotic ancestors through endosymbiosis^[11]^. Plant organellar genomes have markedly contracted during endosymbiotic evolution^[12]^. This contraction is the consequence of loss or endosymbiotic gene transfer (EGT), a special form of horizontal gene transfer (HGT)^[12]^. This is believed to occur principally through the direct movement of DNA^[13]^. In *Arabidopsis thaliana*, 18% of all the nuclear genes are estimated to be of cyanobacterial origin.

Although many varieties of RNA-editing have been reported, only a few systems have been studied in such detail that the editing mechanism is understood and the editing machinery is well-defined^[14]^. Genetic approaches using *A. thaliana* have clarified that the protein family with pentatricopeptide repeat (PPR) motifs are essential for RNA editing in plant chloroplast and mitochondrial^[15]^. Meanwhile, the PPR family has expanded dramatically in plants, and the *A. thaliana* genome encodes approximately 450 members of the PPR family. Some of them possibly bind to the cis-elements of the RNA editing sites to facilitate access of RNA editing enzymes^[16]^.

We, for the first time, reported many details and features of nuclear RNA-editing in a tree species. Our results suggested the acquisition of RNA-editing for the endosymbiont-derived genes in *P. trichocarpa* nuclei is an evolutionary adaptation driven by the endosymbiotic events, which was explicitly demonstrated by the gene ontology enrichment analysis performed on all edited nuclear genes, suggesting RNA-editing plays essential roles for organellar establishment in *P. trichocarpa*. Using a newly developed network approach, we were able to identify a less conserved core motif using C to U edited genes as an example, which may capture the RNA-editing machinery, while enabling large numbers of sites to be targeted.

## RESULTS

### Identification and characterization of RNA-editing sites in *P. trichocarpa* nuclei

**Figure 1:**
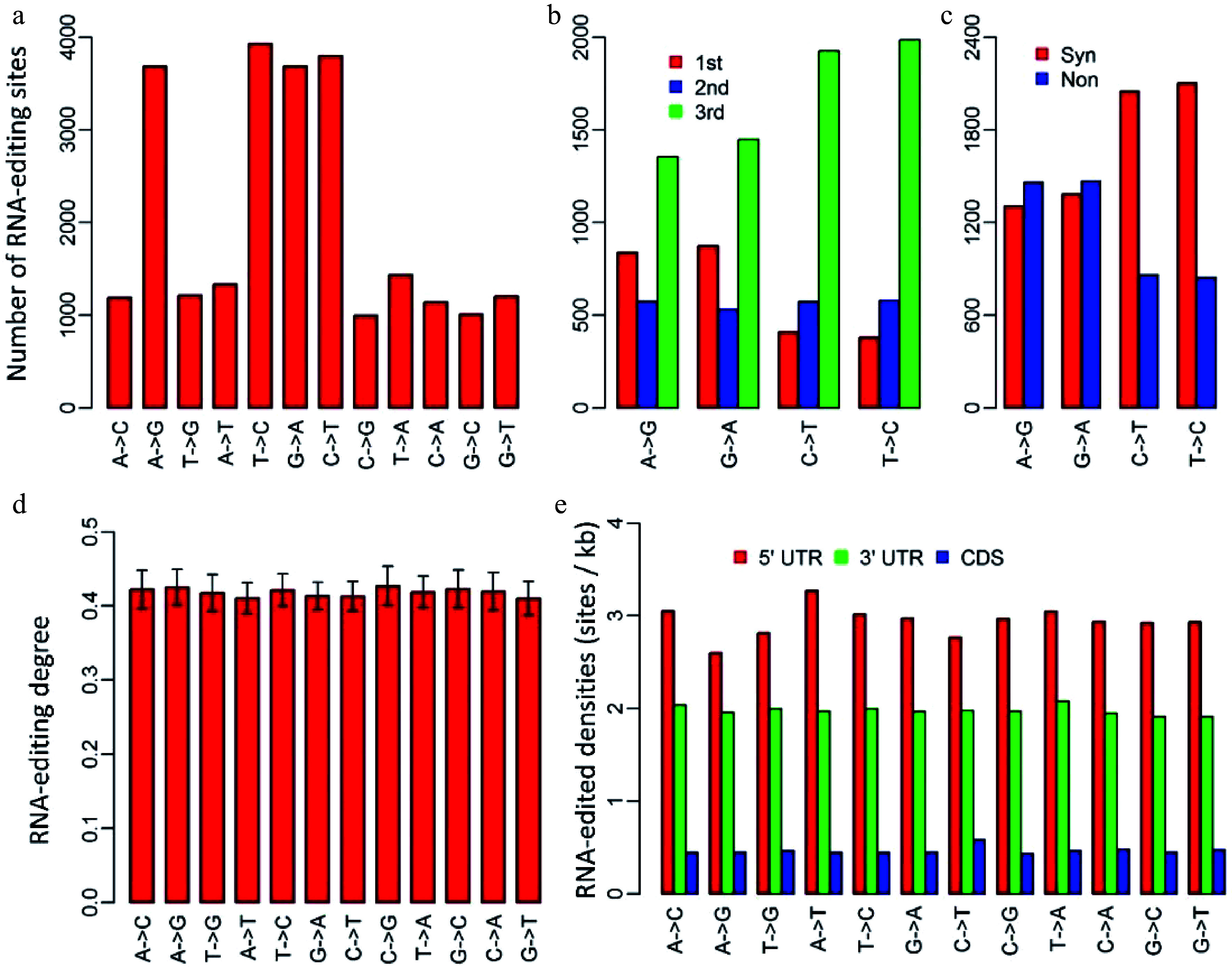
Characteristics of RNA-editing sites in *Populus trichocarpa* nuclear genome. (a) The numbers of the 12 RNA-editing types. (b) The numbers of the four dominant nuclear RNA-editing types in respect to the positions within genetic codons. 1st, 2nd and 3rd represent the first, second and third positions within codons. (c) The numbers of the four dominant nuclear RNA-editing types in respect to synonymous and non-synonymous RNA-editing. Synonymous RNA-editing refers to RNA-editing events that change the RNA sequence but do not change the amino acid sequences, whereas non-synonymous RNA-editing is regarding the events that alter both RNA and amino acid sequences. (d) RNA-editing densities of the 12 RNA-editing types for 5’ untranslated regions (UTR), 3’UTRs and coding regions (CDS). (e) RNA-editing densities of the 12 editing types in respect to 5’UTR, coding regions and 3’ UTRs. CDS represents coding region.

We identified RNA-editing sites in *P. trichocarpa* nuclei using an approach that was modified from the methods used for humans^[17,18]^ and mushroom^[19]^. A total of 24,653 RNA editing sites, located in 8,603 transcripts, were identified. Of these 24,653 editing sites, 2,300, 18,133 and 4,220 were present in 5’-untranslated regions (UTR), coding regions and 3’UTRs, respectively. Though all the 12 RNA-editing types existed in *P. trichocarpa* nuclei, C-to-U, U-to-C, A-to-G and G-to-A were the four primary types, which occupied 61.3% of all the RNA-editing sites (Fig. 1a). This implied that the RNA-editing machineries in *P. trichocarpa* nuclei had a preference for editing types.

We investigated the occurrence of the four dominant RNA-editing types with respect to the positions of targeted bases within genetic codons. Of the 11,506 editing sites that occurred in coding regions, there were 2,769 A-to-G, 2,854 G-to-A, 2,909 C-to-U and 2,974 U-to-C RNA-editing sites (Fig. 1b). Unlike organelles, RNA-editing in *P. trichocarpa* nuclei tended to occur at the third bases of the edited codons, especially for C-to-U and U-to-C types, for which, 66.1% and 67.4% occurred at the third bases of the edited codons, respectively (Fig. 1b). Consistent with this, as much as 70.4% of C-to-U, and 71.3% of U-to-C RNA-editing were synonymous substitutions that did not change the amino acid sequences (Fig. 1c).

Editing degree is an important parameter that was introduced to measure the percentage of the uniquely mapped and edited reads in the total uniquely mapped reads for each RNA-editing site. In *P. trichocarpa* nuclei, editing degrees of the 12 RNA-editing types were quite similar, ranging from 41.0% to 42.7% with standard errors varying from 1.9% to 2.6% (Fig. 1d). Although the averaged editing degrees for the 12 editing types were approximately the same, editing degrees of various transcripts had a considerable variation. For this reason, we used the number of edited transcripts to represent gene expression levels in further analysis (cluster analysis).

To examine if nuclear RNA-editing has a preference for 5’UTRs, coding regions, and 3’UTRs, we calculated editing density for these three regions. The editing density was defined as the number of RNA-editing sites per kilobase. Interestingly, RNA-editing in *P. trichocarpa* nuclei preferred the two types of UTRs rather than coding regions, especially 5’UTRs. Editing densities of 5’UTRs and 3’UTRs among these 12 RNA editing types were about 3 sites (ranging from 2.6 to 3.3) and 2 sites (ranging from 1.9 to 2.1) per kilobase, respectively (Fig. 1e). In contrast, RNA-editing densities of coding regions were only about 0.5 (0.4−0.6) site per kilobase (Fig.1e).

### Transcripts with RNA-editing sites in UTRs appeared to have higher expression levels

**Figure 2:**
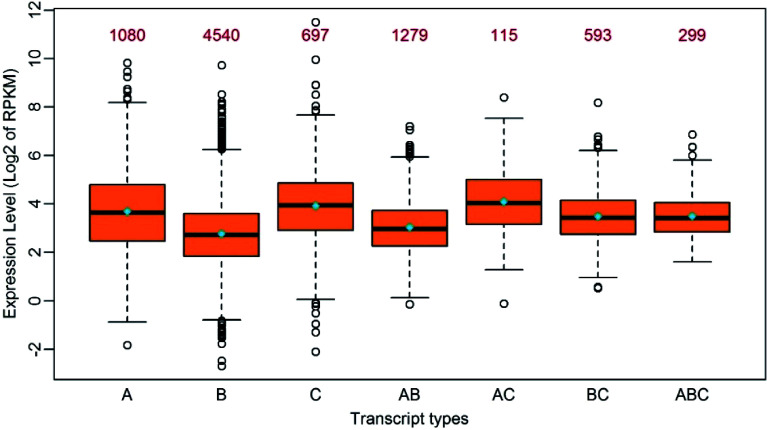
Boxplot of the expression levels of edited transcripts in seven groups. A: Transcripts with RNA-editing only in 5’UTR; B: Transcripts with RNA-editing only in the coding region; C: Transcripts with RNA-editing only in 3’UTR; AB: Transcripts with RNA-editing in 5’UTR and the coding region simultaneously; AC: Transcripts with RNA-editing in 5’UTR and 3’UTR simultaneously; BC: Transcripts with RNA-editing in the coding region and 3’-UTR simultaneously; ABC: Transcripts with RNA-editing in 5’UTR, the coding region and 3’UTR simultaneously. The median and the mean of each group is represented by a horizontal bar and diamond, respectively. The numbers above the boxes represent the numbers of the transcripts in each group.

Given the RNA-editing in *P. trichocarpa* nuclei preference of UTRs rather than coding regions, we investigated if RNA-editing contributed to gene expression to some extent. We classified all the edited transcripts into different groups based on the following criteria: (1) transcripts with RNA-editing exclusively in 5’UTRs, CDS or 3’UTRs were classified into their corresponding groups; (2) transcripts with RNA-editing sites in more than one region were classified into other groups that include 5’UTR-CDS, 5’UTR-3’UTR, CDS-3’UTR and 5’UTR-CDS-3’UTR types. Interestingly, all the transcripts with RNA-editing in 3’UTR, which include 3’UTR, 5’UTR-3’UTR, CDS-3’UTR and 5’UTR-CDS-3’UTR, had higher average expression levels than other types. It is worth noting that the transcripts with RNA-editing in both 5’UTR and 3’UTR at the same time had the highest average expression levels, with a $ \log _2^{RPKM} $ (RPKM, reads per kilobase per million) of 4.1 (Fig. 2). This suggested RNA-editing in 3’UTR had a positive influence on gene expression levels, followed by 5’UTR, and that the effect of RNA-editing sites on gene expression appeared to be additive to some extent.

### RNA-editing occurred with distinct preference for codons corresponding to 20 amino acids and stop codons

**Figure 3:**
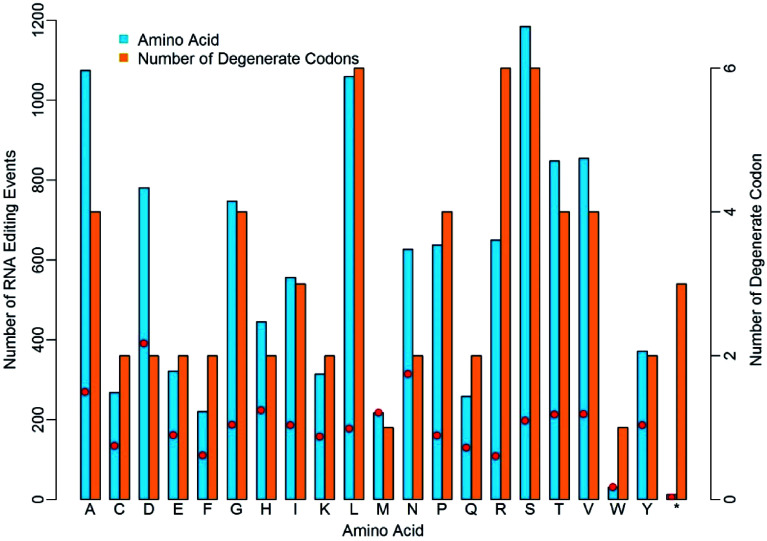
Distinct discrepancy of nuclear RNA-editing events in relation to different amino acids. The red dots represent the ratios of editing sites to the number of degenerate codons.

We investigated the occurrence frequency of RNA-editing in relation to the 20 amino acids in the four dominant RNA-editing types. Considering the degenerate feature of genetic codons, we calculated the ratios of editing sites to the number of degenerate codons for each amino acid. As shown in Fig. 3, RNA editing occurred unevenly to each type of codon that encodes the same amino acid. Obviously, there are more editing events occurring in D, N and A amino acids, while, on the contrary, less editing events occurred in W, F, and R. The amino acids D, N and A had 391, 314 and 269 edited events per codon, whereas W, F, and T had 31, 110 and 108 edited events per codon. The higher or lower editing events on these amino acids suggested the nucleotides corresponding to these codons might entrap or repulse the RNA-editing machinery with distinct discrepancy due to the micro-environment created collectively by hydrogen bonds in these codons^[20,21]^. The RNA-editing on a stop codon is remarkably lower than any other amino acid. The number of degenerate codons ranges from one to six, and there are three stop codons in the standard genetic codon table (UAA, UAG and UGA), however, only 12 of the 11,506 RNA editing events occurred in the stop codons. This was quite low compared to other amino acids.

### The acquisition of RNA-editing for the endosymbiont-origin genes in *P. trichocarpa* nuclei

It is widely accepted that chloroplasts and mitochondria originated from the engulfment of bacteria by an ancestor of the modern eukaryotic cell^[22]^, upon which the genomes of bacteria began to contract dramatically compared to the free-living ancestors of bacteria. The reduction is believed to be a result of HGT. BLAST program was performed to identify the endosymbiont-derived genes in *P. trichocarpa* nuclei. Protein sequences of a cyanobacterium and a α-proteobacterium, the closest relatives of chloroplastic and mitochondrial ancestors, respectively, were scrutinized with an aim to provide a plausible answer. Using a threshold of e-value better than 1e-10, 7,036 genes in *P. trichocarpa* nuclei were considered to be endosymbiont-derived. Of these 7,036 genes, 27.3% (1,922) were subjected to RNA-editing. This percentage is significantly higher than the 19.5% of the non-endosymbiont-derived genes in *P. trichocarpa* nuclei (*P* = 0, Fisher extract test). Was the higher percentage of RNA-editing on these endosymbiont-derived genes inherited from their precursors? If there was RNA-editing in the ancestral cyanobacteria, these endosymbiont-derived genes should, as we showed above, have more codons that could entrap the RNA-editing machinery, and they tended to be edited after being integrated into the plant nuclear genome. To answer this question, we implemented the same procedures for identifying nuclear RNA-editing in poplar to *Synechococcus* sp. PCC 7492, a model cyanobacteria strain. We downloaded 35 RNA-seq data sets from Sequence Read Archive (SRA) database of NCBI (www.ncbi.nlm.nih.gov/sra) to enable this study. Surprisingly, no RNA-editing sites were identified in Synechococcus sp. PCC 7492. This suggested that the RNA-editing of the endosymbiont-derived genes in *P. trichocarpa* nuclei was acquired during endosymbiosis. The higher percentage of endosymbiont-derived genes to be edited in poplar nuclei suggested RNA-editing was, to some degree, acquired in adaptation to fulfill the endosymbiosis.

### Nuclear RNA-editing had a preference for the genes involved in cellular remodeling

**Figure 4:**
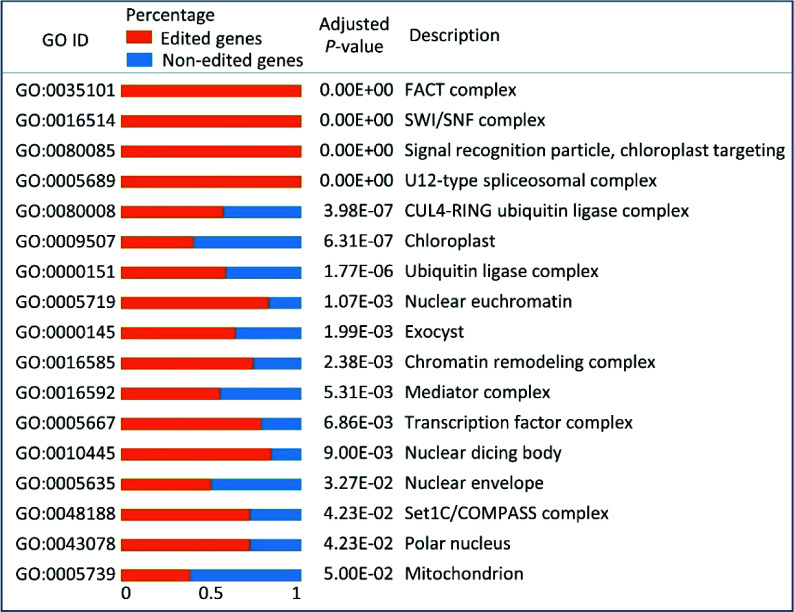
Gene ontology (GO) enrichment results of all the edited genes in *Populus trichocarpa* nuclei. Orange bars and blue bars represent the percentages of edited genes and non-edited genes, respectively, in each GO category.

Gene ontology (GO) enrichment analysis was performed on all the edited genes in an effort to examine which genes had undergone the RNA-editing in *P. trichocarpa* nuclei. A total of 17 GO categories of cellular components were found to be significantly enriched (*p* < 0.05) using hypergeometric distribution (Fig. 4). The GO enrichment result revealed that genes associated with chromatin remodeling, protein degradation, nuclear envelope and organelles were preferentially subjected to RNA-editing. Five chromatin remodeling associated GO categories, including 'chromatin remodeling complex' (GO:0016585), 'nuclear euchromatin' (GO:0005719), 'SWI/SNF complex' (GO:0016514), 'FACT complex' (GO:0035101) and 'Set1C/COMPASS complex' (GO:0048188), which contain 11, 9, 7, 6, and 5 edited genes, respectively. The total genes in these GO categories in the above order are 15, 11, 7, 6 and 7, respectively. In addition, three protein degradation GO categories, that included 'CUL4-RING ubiquitin ligase complex' (GO:0080008), 'ubiquitin ligase complex' (GO:0000151) and 'exocyst' (GO:0000145) were significantly over-represented in the edited genes, which had 75, 61, and 19 genes being edited out of 132, 105, and 30 genes in the background, respectively. Furthermore, 28 genes, 50.0% in the background, which belong to the 'nuclear envelope' (GO:0005635), were subjected to RNA-editing in our GO enrichment results. Additionally, the enriched GO categories, 'chloroplast' (GO:0009507) and 'mitochondrion' (GO:0005739) suggested that RNA-editing in *P. trichocarpa* nuclei had a special preference for genes that were targeted to organelles.

### Identification of genes whose proteins potentially function in RNA-editing machinery in the *P. trichocarpa* genome

One puzzle of RNA-editing is how the edited sites are selected from the large number available. The current model proposes that C-to-U RNA-editing comprises *cis*-elements near the targeted C residues, which can be recognized by PPR proteins that are capable of recruiting unknown editing enzymes^[23]^. 532 homologous *PPR* genes in the *P. trichocarpa* genome were identified using *A. thaliana* PPR proteins as queries in blast analysis. 104 of these PPR proteins had an additional DYW deaminase domain. The DYW is acronym of the three highly conserved C-terminal amino acids: aspartic acid (D), tyrosine (Y), and tryptophan (W), and is known to be essential for cytidine deaminases in some of the DYW PPR proteins, but is not required for functional activity in other PPRs. Two genes (*Potri.017G144900* and *Potri.004G074100*) had an additional RNase_Zc3h12a domain besides PPR domains. Zc3h12a is an RNase essential for controlling immune responses by regulating mRNA decay^[24]^. The large number of PPR family and the divergence of the additional domains in PPR proteins suggested that PPR proteins might play various roles in *P. trichocarpa*.

**Figure 5:**
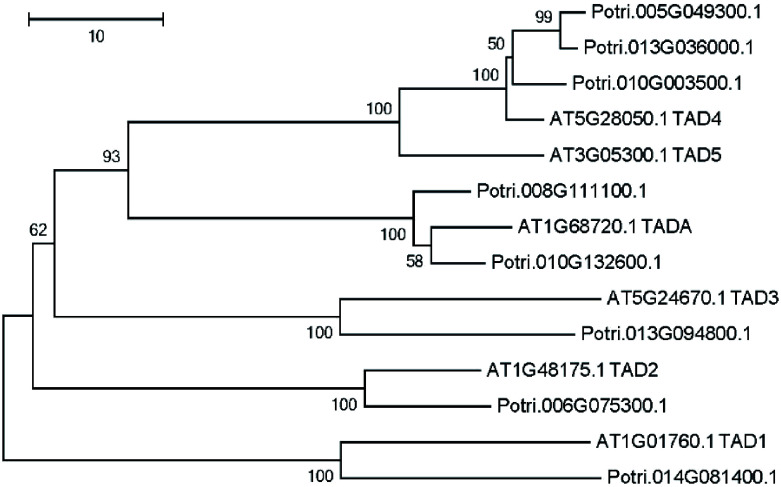
Phylogenetic tree analysis of putative adenosine deaminases in the *Populus trichocarpa* and *A. thaliana* nuclear genome. Protein sequences were aligned with ClustalW, and MEGA was used to construct the phylogenetic tree based on the neighbor-joining method with 1,000 bootstrap replications.

Enzymes that are responsible for Adenosine-to-Inosine (A to I) editing has been identified in *A. thaliana*^[25,26]^. Using these six adenosine deaminases as a query, we identified eight putative adenosine deaminases in the *P. trichocarpa* nuclear genome (Fig. 5). A phylogenetic tree was constructed using all adenosine deaminases in *P. trichocarpa* and *A. thaliana* (Fig. 5). The phylogenetic tree revealed that there were two TADAs in the *P. trichocarpa* nuclear genome, meanwhile, the *A. thaliana* nuclear genome has only one TADA according to a recent report^[25]^.

### Unveiling an A/G-C-A/G motif for C-to-U RNA-editing via cluster analysis of *PPR* genes and C-to-U edited genes

**Figure 6:**
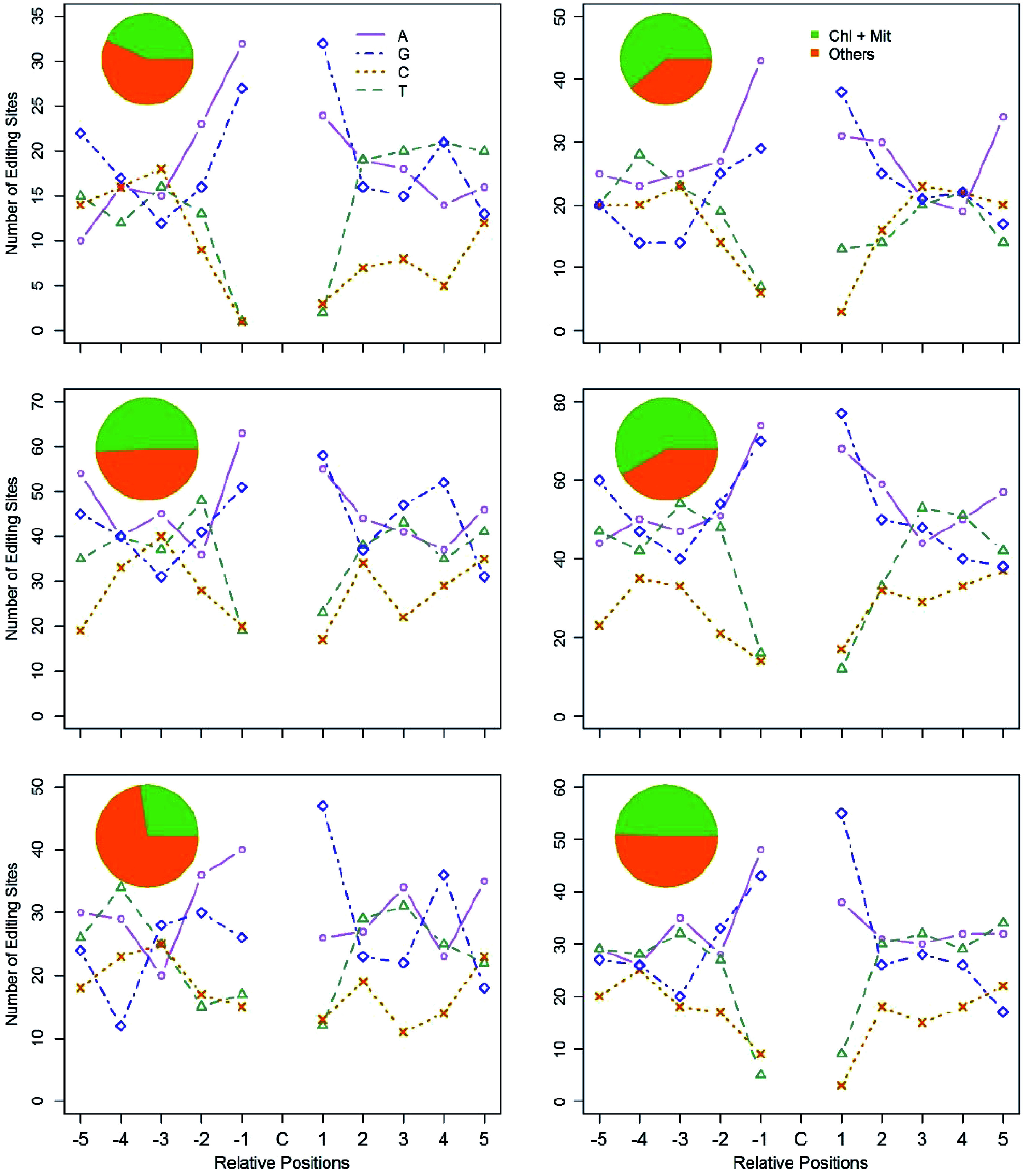
Flanking sequences of the edited C residues, and subcellular location of the edited genes in the six clusters. Using the coexpression based method, we clustered PPR genes and the C-to-U edited genes into six clusters. The pie chart represents the subcellular location of the C-to-U edited genes in each cluster. The line chart represents the ten adjacent bases of the edited C residues in each cluster. For the X axis, C represents the edited C residues; -5 to -1 represent the five upstream bases of the edited C residues; 1 to 5 represent the five downstream bases of the edited C residues. The Y axis represents the number of RNA-editing sites in each cluster.

Recent reports have proven that PPR proteins participate in C-to-U RNA-editing by binding the neighbouring regions of edited sites, especially upstream of the edited nucleotides. However, to date, no consensus conserved motifs have been identified. Since PPRs bind targets directly, we believed that the expression levels of *PPR* genes must be, to some degree, correlated to the edited transcripts of their targets. Based on such a hypothesis, we developed a coexpression based algorithm to group the *PPR* genes and the C-to-U edited genes into 18 clusters (See Materials and Methods section for further detail). The expression levels of the *PPR* genes are more coordinated to the targeted genes within the same cluster. We then examined the flanking nucleotides of the edited sites within each cluster. Surprisingly, out of these clusters, six clusters comprised a total of 46 *PPRs* and 546 RNA-editing target genes that had a consensus motif (Table 1). The −1 and +1 positions relative to the edited C residues were composed primarily of purines (A and G) (Fig. 6), namely, most of the edited C residues were flanked by A/G residues, and the sequence pattern is A/G-C-A/G. As shown in Fig. 6, the pie chart in each plot manifested the percentage of C-to-U edited genes that target organelles in all edited genes in that cluster. One percentage is 27.2% in the fifth cluster, and the other five percentages for the five remaining clusters varied from 43.1% to 60.8%.

**Table 1:** Number of *PPR* genes and C-to-U edited genes in each cluster.

Cluster ID	Number of edited genes	Number of PPRs
1	51	4
2	75	4
3	115	5
4	151	15
5	66	8
6	88	10

### Investigation of RNA-editing sites using expressed sequence tags

**Figure 7:**
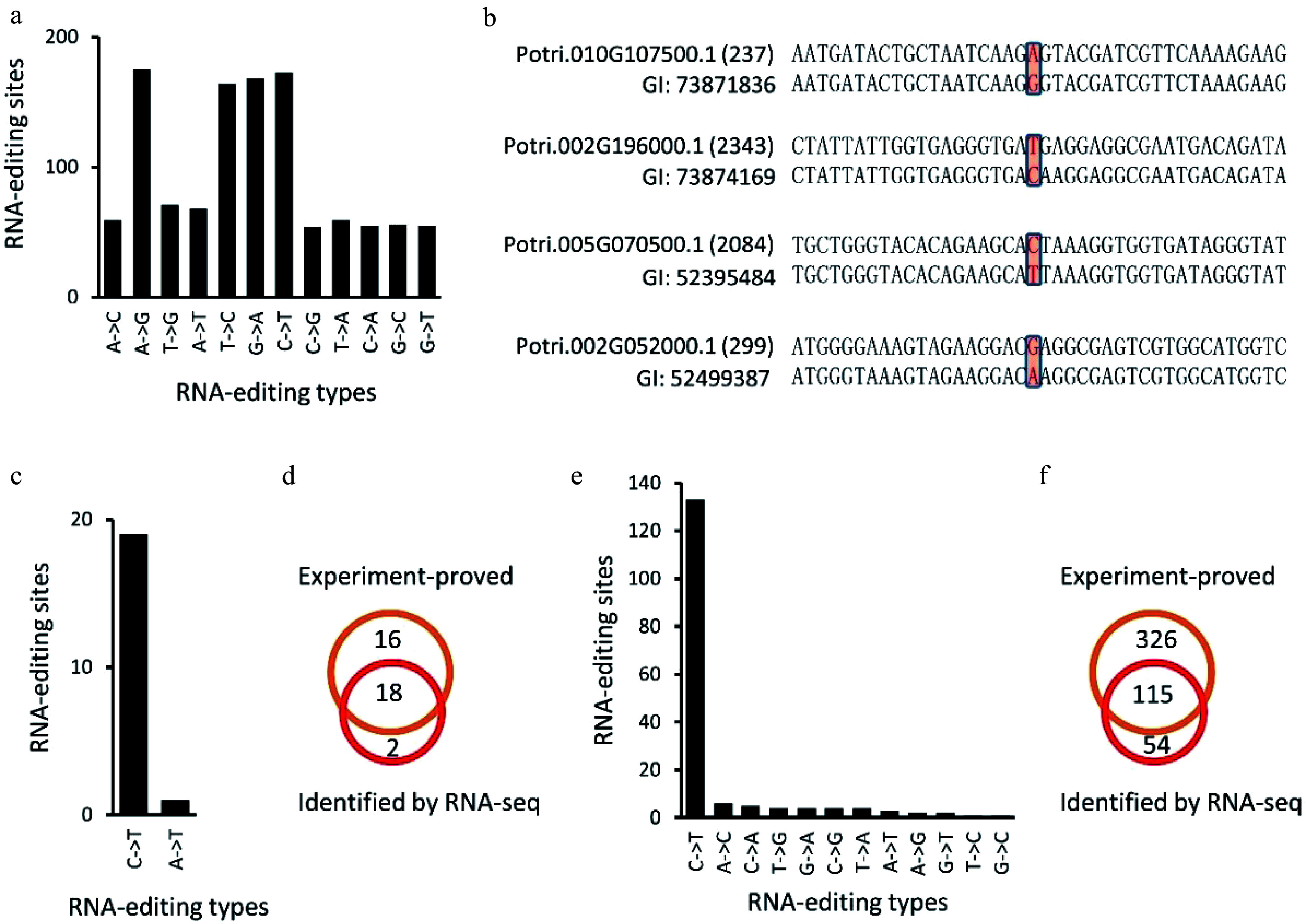
Identification of RNA-editing sites using expressed sequence tags (EST) of *Populus trichocarpa* (Nisqually-1). (a), (b), (e) and (f) showed the identified RNA editing sites in *P. trichocarpa* while (c) and (d) showed the RNA editing sites identified in *A. thaliana* chloroplasts that have been experimentally verified (Fig. 7c, d).

Expressed sequence tags (EST) of *P. trichocarpa* (Nisqually-1) were downloaded from NCBI (ncbi.nlm.nih.gov) to confirm the RNA-editing sites identified in this study. Of these 24,653 RNA-editing sites, 2,171 were covered by ESTs, and these 2,171 editing sites present in 1,272 transcripts. By comparing EST and *P. trichocarpa* genomic DNA sequences of these RNA-editing sites (Fig. 7a−d), we found that 1,157 (53.3%) sites, present in 765 transcripts, were subjected to RNA-editing (Fig. 7a, d). This ratio was even higher than the editing degree, 41.0% to 42.7%.

### Identification of RNA-editing sites in the *A. thaliana* chloroplast genome

Since RNA-editing sites have been well studied in plant chloroplasts^[27]^, we used the *A. thaliana* chloroplast genome (Columbia genotype) to assess the accuracy of our method. The same method was applied to identify RNA-editing sites in the *A. thaliana* chloroplast genome. Using 20 RNA-seq datasets, 20 potential RNA-editing sites were identified in the *A. thaliana* chloroplast genome, including 19 C-to-U and one A-to-U alteration (Fig. 7b, Table 2). Unlike RNA-editing in *P. trichocarpa* nuclei, 18 (90%) of the RNA-editing sites in the *A. thaliana* chloroplast genome occurred on the second position of the codons (Table 2). By comparing these 20 editing sites with the 34 experiment-proved editing sites^[27]^, we found that 18 (90%) of the 20 editing sites have been experimentally confirmed (Fig. 7c, d).

**Table 2:** Identified RNA-editing sites in the *A. thaliana* chloroplast.

Gene	Genome position	DNA	RNA	Position within the codon	Confirmed
atpF ArthCp008	1,207	C	T	2	Yes
rpoB ArthCp014	25,992	C	T	2	Yes
rpoB ArthCp014	25,779	C	T	2	yes
rpoB ArthCp014	23,898	C	T	2	yes
ycf9 ArthCp019	35,800	C	T	2	Yes
rps14 ArthCp020	37,161	C	T	2	Yes
rps14 ArthCp020	37,092	C	T	2	Yes
accD ArthCp031	57,868	C	T	2	Yes
psbF ArthCp038	63,985	C	T	2	Yes
psbE ArthCp039	64,109	C	T	2	Yes
rps18 ArthCp044	67,930	A	T	2	New
clpP ArthCp048	69,942	C	T	1	Yes
rpoA ArthCp055	78,691	C	T	2	Yes
ndhD ArthCp074	117,166	C	T	2	Yes
ndhD ArthCp074	116,785	C	T	2	yes
ndhD ArthCp074	116,494	C	T	2	yes
ndhD ArthCp074	116,290	C	T	2	yes
ndhD ArthCp074	116,281	C	T	2	yes
ndhG ArthCp077	118,858	C	T	2	yes
ndhI ArthCp078	119,549	C	T	1	New

## DISCUSSION

RNA-editing is a post-transcriptional modification of individual nucleotides in RNA molecules. As one of the final steps in RNA pre-processing for maturation, RNA-editing can potentially modulate the expression levels of various RNA species and the corresponding proteins to meet some cellular needs that are still not explicitly defined^[28]^. To date, almost all studies on RNA-editing in plants have been focused exclusively on organellar genomes. As a result, little is known about nuclear RNA-editing. The advent of HTS and sequence analysis software pipelines empowers the study of nuclear RNA-editing in plants. Although the use of RNA-seq data for identifying nuclear-RNA-editing is still in its infancy, a few studies have been reported in humans^[17,29]^, and *A. thaliana*^[9]^. We, for the first time, reported nuclear RNA-editing in a tree species.

Although the use of RNA-seq data to study nuclear RNA-editing is viable, caution needs to be taken regarding some potential pitfalls. One of the noticeable problems is the high false-positive rate of RNA-editing sites that are present in HTS data. The spurious differences between RNA and genomic DNA in individual nucleotides generally originate from three different aspects that include: (i) the use of different genotypes other than the one from which the original genome sequences were derived; (ii) sequencing errors arising from the mistakes in base calling^[30]^; (iii) alignment errors caused by using different alignment algorithms^[31]^. In this study, we used 30 RNA-seq data sets from the same genotype, 'Nisqually 1' of *P. trichocarpa*, which was sequenced by Tuskan et al.^[32]^, for RNA-editing site recognition. In addition, we developed computational pipelines with high stringency to eliminate the false positive rate caused by sequencing and alignment errors (See the Materials and Methods for further details). We integrated more strict thresholds into our pipelines, which include the requirement of at least 50 times coverage of high-quality reads, 100% matches in seeds (22 nt), and at most three candidate editing sites allowed in the final aligned reads. These strict thresholds were able to reduce the false-positive sites significantly and led to the identification of candidate RNA-editing sites. Furthermore, Fisher’s exact test was employed to compare the observed and expected difference between RNA and its genomic DNA. A confidence level of 0.05, corrected by false discovery rate^[33]^ was used to determine the statistical significance of each potential editing site.

### Acquisition of nuclear RNA-editing on endosymbiont-derived genes upon endosymbiosis

Mitochondria and chloroplasts are known to be evolved from the α-proteobacterial and cyanobacterial ancestors via endosymbiosis^[34]^, during which, the majority of endosymbiotic genes have been integrated into the host genome through HGT^[12,35]^. Our study revealed that at least 17% of *P. trichocarpa* nuclear genes are of endosymbiont-origin. This percentage was consistent with that of *Arabidopsis* (> 18%)^[36]^. Our results showed 27.3% of the transcripts of the endosymbiont-derived genes were edited, whereas only 19.5% of the transcripts of other genes were modified. Why did nuclear RNA-editing tend to modify endosymbiont-derived genes? We further investigated RNA-editing in a cyanobacterium using public data and the same methodologies as we implemented for *P. trichocarpa*. Surprisingly, there was no RNA-editing at all in this bacterium. In addition, there is no evidence in the existing literature that implies there is RNA-editing in either α-proteobacteria or cyanobacteria. These facts indicate that RNA-editing of the endosymbiont-derived genes was acquired during endosymbiosis. This conclusion is in agreement with recent studies on replacement plastids in dinoflagellates^[37]^, where RNA-editing has been acquired upon the replacement plastids being present.

### RNA-editing in *P. trichocarpa* nuclei preferentially occurred on the endosymbiont-derived genes and intracellular remodeling genes in adaptation to establishment of endosymbiosis

**Figure 8:**
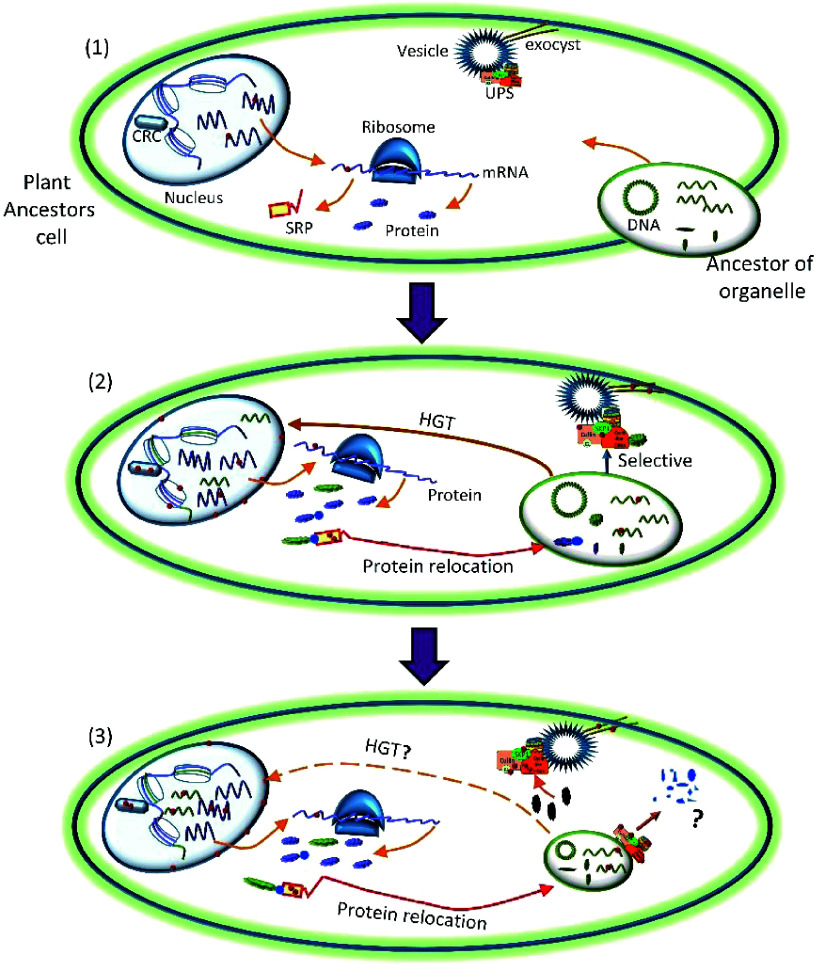
A model of the establishment of plant organelles and possible roles of RNA-editing during this process. Protein complexes or cellular components with red dots indicated they were subjected to RNA-editing. At the beginning of endosymbiosis (1), the plant ancestral cell engulfed a prokaryotic bacterium. (2) Upon engulfment, plant ancestral cells performed significant intracellular remodeling, which altered the nuclear membrane, the protein degradation system and the chromatin remodeling system, to embrace this bacterium. (3) After successful establishment of organelles, the genome of the organelle was significantly smaller compared to its ancestor. HGT: horizontal gene transfer. CRC: chromatin remodeling complex. UPS: ubiquitin-proteasome system. SPR: signal recognition particle, chloroplast targeting.

Are certain types of genes preferentially edited by nuclear RNA-editing machinery? Do they have particular functions? To answer these questions, GO enrichment analysis of the edited genes was performed, the results demonstrated several GO categories that contained genes involved in protein degradation were particularly enriched. These included 75 (56.8%) genes of CUL4-RING ubiquitin ligase complex and 61 (58.1%) genes of ubiquitin ligase complex, which suggested that RNA-editing occurred in the ubiquitin-proteasome system (UPS) to specifically facilitate the establishment of organelles during endosymbiosis. As shown in Fig. 8, at the beginning of endosymbiosis, the plant ancestral cells need to perform significant remodeling in order to adopt the endosymbionts for mutual beneficial determination rather than destroy and/or expel them^[38]^. To achieve this, plant ancestors were obligated to modify their UPS to mediate the degradation of useful proteins produced by endosymbionts. This is also supported by the fact that the existing UPS is actually present at the outer membrane of both mitochondria and chloroplasts where they mediate ubiquitination and degradation of organellar proteins^[39]^, suggesting that RNA-editing may contribute to UPS’s dynamic regulation on organellar functionality through modifying its own components at the RNA level as UPS genes are known to be one of the conserved genes at the DNA level. A further two recent studies have shown that E3 ubiquitin ligases act as a regulator of symbiosis receptor kinase, and are involved in rhizobial infection and nodulation in *Lotus japonicas*^[40]^. All these pieces of evidence suggest UPS play essential roles in the process of symbiosis. Besides the remodeling of the protein degradation system, the plant ancestral cells might also modify their exocytosis systems through RNA-editing to avoid the expulsion of whole endosymbionts, and/or discharge unwanted or 'trouble-maker' protein products from endosymbionts. This is demonstrated by the overrepresented genes of the exocyst complex, 19 (63.3%) genes in this GO category were subjected to RNA-editing. The evidence for the important role of the exocytosis system in symbiosis has been shown in some studies, for example, the exocyst complex was shown to play a role in expulsing the symbionts in modern experimental systems^[41]^, *Rhizobium*–legume symbiosis shares an exocytotic pathway required for arbuscule formation^[42]^ and rapid exocytosis plays a role in symbiotic interaction of algae and a sea anemone at low temperatures^[41]^ (Fig. 4).

To conquer the endosymbionts, plant ancestral cells appeared to employ nuclear RNA-editing to facilitate HGT (Fig. 8). This is reflected by the significant modification of the nuclear membrane associated genes. In our results, 28 (50%) 'nuclear envelope' related genes were subjected to RNA-editing. These edited genes might affect the permeability of the nuclear membrane of the host cells and thus facilitate the entry of endosymbiotic genes into its host nuclei. This is a crucial step for HGT. In addition, after successfully taking over the genes from endosymbionts, the requirement of spatio-temporal expression of the integrated genes in plant genome necessitated the establishment of the chromatin structure. RNA-editing appeared to be involved in this process too. This is evidenced by the enrichment of the genes involved in chromatin remodeling and linked processes. For example, 11 (73.3%) genes in the 'chromatin remodeling complex', 7 (100%) in the 'SWI/SNF complex', 6 (100%) in the 'FACT complex', 5 (71.4%) in the 'Set1C/COMPASS complex', and 9 (81.8%) in 'nuclear euchromatin' were collectively enriched (Fig. 4).

To be mutually beneficial, many proteins translated from the endosymbiont-derived genes and some nuclear genes, were labeled with a 'signal peptide' that enables them to work in organelles to govern and/or maintain organellar functions. In our results, 40.4% and 38.3% of genes whose products targeted the chloroplasts and mitochondria are enriched among RNA-editing targets, respectively, and three (100%) genes involved in the 'signal recognition particle, chloroplast targeting' were enriched (Fig. 4). This suggested that RNA-editing plays some roles in modification and accurate subcellular localization of these 'regulator and worker' proteins.

### RNA-editing in *P. trichocarpa* nuclei tended to be synonymous substitutions

In contrast to RNA-editing in organelles that usually target at the first (~30%) or second (~58%) bases of codons^[43]^, RNA-editing in *P. trichocarpa* nuclei tended to occur at the third bases of codons, and thus did not, in general, change amino acids. Why did nuclear RNA-editing evolve such a mechanism to modify the third base of a codon? Although the explicit answer for this is still elusive, one early study indicated that nuclear RNA-editing also occurs to the first and second positions of codons in a higher frequency, but nuclear RNA-editing can cause more serious deleterious effect in genomes, and as a result, the mutants caused by the RNA-editing on the first or second base of codons are likely to be eliminated due to impairing normal functions or adaptation^[44]^. However, such an explanation is obviously contradictory with RNA-editing in organelles. Why it is not detrimental in organelles? Our explanation for this is that there is less selection pressure in organelles. In nuclei, genes have a complicated wired network, the effect caused by one node can propagate in the gene network and affect large number of genes, resulting in more mutants dieing. We postulate that the nuclear editing is a passive mechanism that can avoid large numbers of nuclear genes being mutated. This is evidenced by our result that 59.6% RNA-editing in the coding region were synonymous modifications. For the 40.4% non-synonymous modifications, it may be evolutionally important for producing functionally diversified proteins to deal with endosymbiosis.

### PPR proteins participated in C-to-U RNA-editing in *P. trichocarpa* nuclei by binding an A/G-C-A/G *cis*-element that confers wide targeting

It has been widely reported that PPR proteins directly bind the flanking nucleotides of the edited sites in C to U conversions^[45]^. However, so far, no consensus motifs have been reported. To explore this problem, we used a co-expression network-based approach to cluster the *PPR* genes and all the C-to-U edited genes in *P. trichocarpa* nuclei into different groups. We hypothesized that expression of *PPR* genes and their modified RNA species should have a predator and prey relationship as PPR proteins directly bind the targeted RNAs and process them into mature RNAs. Surprisingly, we were able to identify a core motif, A/G-C-A/G, in all the six clusters that were obtained from the decomposition of the coexpression based network, suggesting RNA-editing can, to some degree, affect the coordination of their target gene expression. The percentages of −1 and +1 bases around C residues [A/G] in these six clusters (in total 546 genes) varied from 67.3% to 96.7%, and the average percentages of these two positions [A/G] were 78.9% and 79.9%, respectively. The Chi-square tests of these two positions’ composition in comparison to the background matrix in all six clusters were all above the significant levels. Therefore, [A/G]C[A/G] probably served as the core motif for entrapping PPR to C residues. The further up- or down-stream nucleotides of C residues in the 546 genes of these six clusters were not conserved, suggesting that there was no highly conserved longer motif for RNA-editing machinery to recognize. At first glance, this may appear disappointing, however, a conserved shorter core motif can enable PPR to target large numbers of sites. Further study is needed to verify such a conclusion.

## MATERIALS AND METHODS

### Data preparation

RNA-seq reads of *P. trichocarpa*, *Synechococcus elongatus* PCC 7942 and *A. thaliana* were downloaded from the Sequence Read Archive (SRA) database of NCBI (www.ncbi.nlm.nih.gov/sra). The *P. trichocarpa* RNA-seq datasets used in this study are SRP035471 and SRP028843. These two data sets have a total of 30 RNA-seq libraries, and the genotype for the poplar RNA-seq data is Nisqually-1, the same clone as the sequenced poplar^[32]^. SRP035471 and SRP028843 are single end reads with a length of 68 bp and 100 bp, respectively. These two datasets contain 7.5 Gb and 23.1 Gb data, respectively. Materials used for SRP035471 and SRP028843 are developing xylem and stem differentiating xylem, respectively. The RNA-seq datasets of *S. elongatus* PCC 7942 are SRP030395 and SRP020509, which contain 18 and 17 single-ended RNA-seq datasets, respectively. The RNA-seq data used for identifying RNA-editing in *A. thaliana* chloroplasts and mitochondria is SRP036525, which contains 24 single-ended RNA-seq datasets. All of the SRA data were converted in to fastq format using fastq-dump, a utility contained in SRA Toolkit.

The latest *P. trichocarpa*^[32]^ genome sequence was download from phytozome v10 website^[46]^. The genome sequence of *S. elongatus* PCC 7942 was download from NCBI genome database (www.ncbi.nlm.nih.gov/assembly/GCF_000012525.1). The mitochondrial and chloroplast sequences of *A. thaliana*^[47,48]^ were download from TAIR^[49]^. The protein sequences used as queries for identification of organelle-derived genes in *P. trichocarpa* nuclei were download from NCBI. The accession numbers of the cyanobacterium and alpha-proteobacterium are NC_007604.1 and YP_006757382.1.

### Short Reads Mapping and RNA-editing sites identification

The use of RNA-seq data to study nuclear RNA-editing has been conducted in humans^[17]^, and mushroom^[19]^. We used the same approach with some modifications to investigate RNA-editing in *P. trichocarpa* nuclei. The primary transcripts of *P. trichocarpa* were used as templates for alignment. All of the RNA-seq reads were aligned to the templates using bowtie2, version 2.2.1^[50]^, with perfect matches being required for seed alignments of 22 nt, and no more than three candidate editing sites allowed in finally aligned reads, and an end to end alignment format was used. The SAM alignment result was converted to BAM format, and then indexed and sorted using Samtools 0.1.18^[51]^. The single nucleotide variants (SNVs) were called using the mpileup tool that was integrated in Samtools. SNVs were further filtered as follows. First, only SNVs with at least 50 high quality raw reads were retained for further analysis. Secondly, Fisher’s exact test was employed to determine if the DNA-RNA differences were authentic events or sequencing errors. Multiple test (FDR method) was introduced to adjust the p-values^[33]^. Only variants with adjusted *p-values* less than 0.05 were considered as RNA-editing sites. After identification of RNA-editing sites, perl scripts were developed to characterize these editing sites.

### Identification of endosymbiont-derived and *PPR* genes in the *P. trichocarpa* nuclear genome

BLASTP with a cutoff of evalue < 1E-10 was performed to identify endosymbiont-derived and *PPR* homologous genes in the *P. trichocarpa* nuclear genome according to previous reports^[36,52]^. Proteins of *S. elongatus* PCC 7942 and alpha-proteobacterium HIMB59 were used as queries for the identification of endosymbiont-derived genes. All the *A. thaliana* pentatricopeptide repeat (PPR) proteins predicted by Claire Lurin et al. were used as a query for PPR prediction in *P. trichocarpa*^[53]^. For identification of PPR genes, pfam scan and *batch* Web CD-Search Tool were employed for domain prediction^[54,55]^. Proteins without significant PPR motifs were discarded.

### Clustering of C-to-U edited transcripts and *PPR* genes

We constructed an expression data sets of *PPR* genes and the C-to-U edited-genes. For these edited-genes, the expression levels were adjusted using editing degrees. The editing degree was calculated as the ratio of uniquely mapped RNA-reads that contain edited nucleotides to total uniquely mapped ones. For transcripts with more than one editing site, the average editing degrees were used for adjustment. Spearman rank co-expression analysis was then applied to the data set, and a coexpression/coordination network represented by Shared Coexpression Matrix (SCM) was built. The SCM was then decomposed into clusters according to published methods^[56]^. The gene pair (*G*_*i*_-*G*_*j*_) that shared the highest coexpression strength was chosen as a primer. The third gene (*G3*) with a significant number of shared coexpressed genes with *G*_*i*_ and *G*_*j*_ was added into the cluster if it met the required constraints of coexpression. Next, all genes with a significant number of shared coexpressed genes, with at least three genes that were already in the cluster, were added. A cluster was produced when no more genes could be added. All genes in this cluster were then removed from SCM before the next round analysis was initiated. After generating of clusters, we examined the genes in each cluster. A perl script was then developed to extract the flanking sequences of editing sites in each cluster. To identify if there are any conserved motifs in the clustered genes, we cut the −5 and +5 flanking sequence of the C residue in all transcripts of each cluster, and then built a two-dimensional composition matrix for each cluster. One dimension is a possible base composition (A, T, C, and G), the other dimension is the positions around the C-residue. The C-residue had a position of zero. The upstream positions were named sequentially as −1, −2, −3, …, −5 whereas the downstream positions of C-residue were named in order as +1, +2, +3, …, +5. At the same time, we built a background matrix around the C residue using all primary transcript sequences in poplar. We then used Chi-square test to check which composition at each given position was significant between a matrix built from a cluster and the background matrix.
